# Comprehensive Metabolomic Profiling of Cord Blood and Placental Tissue in Surviving Monochorionic Twins Complicated by Twin-Twin Transfusion Syndrome With or Without Fetoscopic Laser Coagulation Surgery: A Retrospective Cohort Study

**DOI:** 10.3389/fbioe.2022.786755

**Published:** 2022-04-12

**Authors:** Tianjiao Liu, Li Wen, Shuai Huang, Ting-li Han, Lan Zhang, Huijia Fu, Junnan Li, Chao Tong, Hongbo Qi, Richard Saffery, Philip N. Baker, Mark D. Kilby

**Affiliations:** ^1^ State Key Laboratory of Maternal and Fetal Medicine of Chongqing Municipality, Chongqing, The First Affiliated Hospital of Chongqing Medical University, Chongqing, China; ^2^ International Collaborative Laboratory of Reproduction and Development, Ministry of Education, Chongqing Medical University, Chongqing, China; ^3^ Department of Obstetrics, The First Affiliated Hospital of Chongqing Medical University, Chongqing, China; ^4^ Department of Reproduction Health and Infertility, The First Affiliated Hospital of Chongqing Medical University, Chongqing, China; ^5^ Chongqing Women and Children’s Health Center, Chongqing, China; ^6^ Cancer, Disease and Developmental Epigenetics, Murdoch Children’s Research Institute, Parkville, VIC, Australia; ^7^ Department of Pediatrics, University of Melbourne, Parkville, VIC, Australia; ^8^ College of Life Sciences, University of Leicester, Leicester, United Kingdom; ^9^ Institute of Metabolism and System Research, University of Birmingham, Birmingham, United Kingdom; ^10^ Fetal Medicine Centre, Birmingham Women’s and Children’s Foundation Trust, Birmingham, United Kingdom

**Keywords:** twin-twin transfusion syndrome, fetoscopic laser coagulation, metabolomics, cord plasma, placenta

## Abstract

**Objectives:** To investigate metabolomic perturbations caused by twin-twin transfusion syndrome, metabolic changes associated with fetoscopic laser coagulation in both placental tissue and cord plasma, and to investigate differential metabolites pertinent to varying fetal outcomes, including hemodynamic status, birth weight, and cardiac function, of live-born babies.

**Methods:** Placental tissue and cord plasma samples from normal term or uncomplicated preterm-born monochorionic twins and those complicated by twin-twin transfusion syndrome treated with or without fetoscopic laser coagulation were analyzed by high-performance liquid chromatography metabolomic profiling. Sixteen comparisons of different co-twin groups were performed. Partial least squares–discriminant analysis, metabolic pathway analysis, biomarker analysis, and Spearman’s correlation analysis were conducted based on differential metabolites used to determine potential biomarkers in different comparisons and metabolites that are pertinent to neonatal birth weight and left ventricular ejection fraction.

**Results:** These metabolomic investigations showed that the cord plasma metabolome has a better performance in discriminating fetuses among different hemodynamic groups than placental tissue. The metabolic alteration of twin-twin transfusion syndrome in these two types of samples centers on fatty acid and lipid metabolism. The fetoscopic laser coagulation procedure improves the metabolomic change brought by this syndrome, making the metabolomes of the treated group less distinguishable from those of the control and preterm birth groups. Certain compounds, especially lipids and lipid-like molecules, are noted to be potential biomarkers of this morbid disease and pertinent to neonatal birth weight and ejection fraction.

**Conclusions:** Fetoscopic laser coagulation can ameliorate the metabolomic alteration caused by twin-twin transfusion syndrome in placental tissue and cord plasma, which are involved mainly in fatty acid and lipid-like molecule metabolism. Certain lipids and lipid-like molecules are helpful in differentiating co-twins of different hemodynamic statuses and are significantly correlated with neonatal birth weight or ejection fraction.

## Introduction

Perinatal mortality and morbidity are three to seven times higher in twin pregnancy than in singleton pregnancy (Hack et al., 2008). In monochorionic diamniotic (MCDA) twin pregnancies, which are prone to multiple concomitant complications such as twin-twin transfusion syndrome (TTTS), selective intrauterine growth retardation (sIUGR), severe weight discordance, and twin anemia-polycythemia sequence (TAPS), the perinatal mortality rate is even higher (11%) (Hack et al., 2008; Lewi et al., 2008); thus, more insights into this field are desperately needed. Among these complications, TTTS, mainly caused by unidirectional intertwin blood flow *via* placental arteriovenous anastomoses (AVA), is almost the most detrimental. It could result in severe hypovolemia and anemia in the donor fetus and serious hypertensive hemodynamic perturbation in the recipient twin’s circulation, with subsequent myocardial hypertrophy and even cardiomegaly in the later stage (Stirnemann et al., 2010). TTTS complicates approximately 9–15% of MCDA pregnancies (Sebire et al., 2000; Lewi et al., 2008) and accounts for approximately half of the stillbirths in MCDA pregnancies (Lewi et al., 2008). For those with onset in the second trimester, if left untreated, this syndrome is highly fatal for each fetus (at least 90%) (Van Mieghem et al., 2009), and surviving fetuses are at high risk of suffering from many undesirable perinatal outcomes and sequelae, such as preterm births (PTBs) (Ong et al., 2006), severe cardiovascular anomalies (Michelfelder et al., 2015; Pruetz et al., 2015), neurological impairments (van Klink et al., 2016), renal dysfunction (Melhem et al., 2019), and fetal endocrinal dysregulation (Bajoria et al., 2002). If onset occurs in late pregnancy, a timely cesarean section is recommended to ensure the survival of both twins, though the aforementioned adverse outcomes may also occur (Simpson and Simpson, 2013).

Recent decades have witnessed the advent and advancement of invasive therapeutic techniques for curing TTTS (Senat et al., 2004; Ruano et al., 2013; Slaghekke et al., 2014). Fetoscopic laser coagulation (FLC) aims to photocoagulate the pathological AVA between co-twins on the surface of the monochorionic placenta and therefore restores hemodynamics in the circulation of both fetuses. With the survival rate of both co-twins approaching 70% and the noticeable reduction in the occurrence of neurodevelopmental morbidity and cardiac dysfunction (Senat et al., 2004; Ruano et al., 2013; Diehl et al., 2017), FLC has grossly outperformed serial amnioreduction in improving the prognosis of twins complicated by TTTS, making it the current first-line effective therapy for TTTS (Senat et al., 2004).

Metabolomics is the full-scale untargeted deciphering of the metabolic profile of biological samples. The metabolome, resulting from the interplay of genotype and the environment, can provide much critical and sensitive bioinformation on the pathophysiology of human disease and physiological activities. Given that the molecular mechanism that drives the occurrence of communicating AVA in the MCDA placenta is poorly understood and an animal model for the study of TTTS is lacking, metabolomics could be applied as a promising tool in TTTS research. However, notwithstanding the considerable merits of metabolomic studies, due to the low overall incidence of TTTS and the difficulty in retrieving biological samples, very little evidence has been reported to elucidate metabolomic disturbances in twins suffering from TTTS, not to mention those who underwent FLC procedures. In our analysis of maternal plasma, our laboratory previously found that TTTS-complicated pregnant women had an altered metabolome before 16 weeks of gestation, and these alterations included changes in fatty acids, Krebs cycle intermediates, and amino acids (Yang et al., 2021). Correspondingly, it was previously implied that the altered carbohydrate and fatty acid profile in the amniotic fluid of the TTTS-complicated recipient co-twin has a close association with FLC treatment, fetal cardiovascular dysfunction, and anomalies (Dunn et al., 2016). However, to date, the metabolomics of samples which are more relevant to the fetal circulation in TTTS with or without FLC surgery has rarely been reported.

In this research, we applied holistic metabolomic fingerprinting, by LC-MS (liquid chromatography–mass spectrometry), of placental tissue and umbilical cord blood plasma collected from normal MCDA pregnancies, apparently uncomplicated preterm-born MCDA twins, TTTS twins treated with FLC surgery, and TTTS pregnancies that were untreated due to the late onset of the syndrome to delineate the metabolomic alterations caused by TTTS and metabolic improvement conferred by the FLC operation and to identify significant metabolite correlations with the discrepancy in neonatal birth weight and ejection fraction within 2 days after birth.

## Materials and Methods

### Participants

**FIGURE 1:**
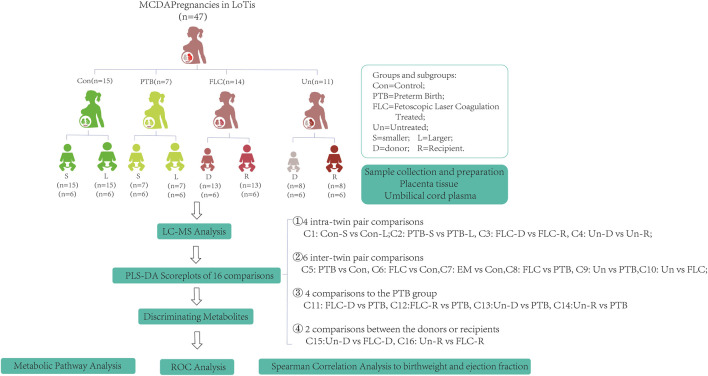
Schematic diagram of the study design. The abbreviations are as follows: MCDA, monochorionic diamniotic; TTTS, twin-to-twin transfusion syndrome; Con, control; FLC, fetoscopic laser coagulation; Un, untreated; PTB, preterm birth; C, comparison; S, smaller twins; L, larger twins; D, donor twins; R, recipient twins.

As shown in Figure 1, 15 normal MCDA twin pregnancies, 25 MCDA twin pregnancies complicated by TTTS (14 FLC-treated cases and 11 untreated cases), and seven preterm-born MCDA twin pregnancies were retrospectively selected from an ongoing twin cohort study conducted at the Department of Obstetrics at the First Affiliated Hospital of Chongqing Medical University. These groups are abbreviated as Con, PTB, FLC, and Un groups in this article, and the 16 comparisons between groups or co-twins are abbreviated as C1–C16 (Figure 1). The identification of chorionicity was conducted by ultrasonic monitoring at 11–14 weeks’ gestational age and confirmed by postpartum examination. Thereafter, MCDA twin pregnancies were followed up by routine ultrasound monitoring every 2 weeks. According to the internationally recognized guidelines, TTTS was diagnosed when the following ultrasound criteria were met: the presence of oligohydramnios (defined as a maximal vertical pocket (MVP) <2 cm) in one amniotic sac and polyhydramnios (an MVP >8 cm before the 20th gestational week (GW) or >10 cm after the 20th GW) in the other sac (Simpson and Simpson, 2013). The staging of TTTS was conducted with the observance of the Quintero system based on sonographic findings (Quintero et al., 1999). TTTS neonates were categorized as larger (or recipient) co-twins and smaller (or donor) co-twins. Because most TTTS pregnancies are complicated by preterm delivery, we included preterm MCDA without apparent fetal complications. The cases in the untreated group had late but rapid onset (>28 weeks when diagnosed with TTTS), so they were promptly administered with dexamethasone, underwent cesarean section on the third day after diagnosis (Supplementary Table S1), and did not receive the FLC procedure due to safety concerns (Simpson and Simpson, 2013). Cases in the control MCDA twin group had appropriate fetal development and a delivery age beyond 37 GWs. The PTB and control groups were managed during the same time span as the TTTS group. All twin pregnancies in the present study were delivered by a cesarean section, and monochorionicity, integrity, and pathological AVA of the placenta were promptly checked by experienced midwives or obstetricians at delivery.

### Fetoscopic Laser Coagulation and Postoperative Follow-Up

FLC surgeries were performed by two experienced operators from the Department of Obstetrics at the First Affiliated Hospital of Chongqing Medical University. The selective sequential FLC technique was adopted in most cases, and an additional “Solomon” procedure was applied in the remaining cases, as described in some published reports (Senat et al., 2004; Morris et al., 2010; Slaghekke et al., 2014). The clinical information of the FLC-treated patients is presented in Supplementary Table S1. Postoperative ultrasound examinations for all participants were performed every week during follow-up. Dexamethasone was prophylactically administered at approximately 32 GWs for subjects with viable fetus (es) to promote fetal lung maturation. All viable pregnancies were advised to be delivered by cesarean section at the 34th to 36th GW if no undesirable complications occurred.

### Echocardiography of Neonates

The echocardiographic monitoring of newborns in this study was performed within 2 days of birth and conducted according to previous studies and published clinical guidelines (Mertens et al., 2011; Tissot et al., 2018; Phad and Waal, 2020) by several experienced ultrasound specialists. LV endocardial tracing was performed semiautomatically using speckle tracking software. All parameters were measured three times and are shown as averages. LV-EF was calculated according to the following formula: EF (%) = SV/EDV × 100, where EF = ejection fraction, SV = stroke volume, and EDV = end-diastolic volume. The SV was calculated as the left ventricle EDV minus the left ventricle end-systolic volume (ESV).

### Sample Collection and Metabolite Extraction

The umbilical cord plasma and placental tissue samples were immediately collected when the placenta was delivered. The demarcation of the monochorionic placenta was conducted according to the septum of the amniotic membrane and the distribution of different placental vasculatures in the co-twins. We collected placental tissues that were approximately 0.5 × 0.5 × 0.5 cm^3^ adjacent to the individual insertion point of the umbilical cord from the fetal side. Placental tissue was instantly washed in precooled physiological saline to remove blood clots, blotted dry, and then frozen in liquid nitrogen for further use. Four milliliters of blood was drained from each umbilical vein and transferred into EDTA-coated blood collection tubes, which were later centrifuged at 3,000 rpm for 10 min at 4°C. The supernatant was aliquoted into cryopreservation tubes (Micronic, Holland) and then stored at −80°C prior to analysis. Fifty milligrams of pretreated placental tissue was mixed with 20 μl internal standard (fexofenadine hydrochloride, 50 μg/ml), 300 μl methanol, and a tiny iron bead in a 2-ml Eppendorf tube, sealed with a sealing membrane, and then homogenized in a tissue disruptor (QIAGEN^@^, TissueLyser II) for 5 min at 25 r/s. The supernatant was transferred into a new set of 1.5-ml Eppendorf tubes. A total volume of 100 μl cord plasma was vortex-mixed with 20 μl of the aforementioned internal standard for 15 s and then with 600 μl acetonitrile for 30 s. Each sample was centrifuged at 12,000 rpm for 15 min at 4°C, dried under a vacuum with a CentriVap^®^ concentrator (Labconco, Kansas City, MO, United States) at room temperature for 7 h to induce metabolite stability, and cryopreserved at −80°C until further analysis.

### Ultra-Performance Liquid Chromatography–Mass Spectrometry (UPLC-MS) Analysis

A metabolomic analysis of the placental tissue and umbilical cord plasma extracts was conducted, as previously described (Wen et al., 2019; Chen et al., 2020; Wu et al., 2021). Each placental tissue sample was reconstituted in 100 μl of acetonitrile water mixture (1:1, v/v), vortexed, and centrifuged at 12,000 rpm for 5 min, and then 100 μl of supernatant was injected into a loading vial for metabolomic fingerprinting. Similarly, each cord plasma sample was vortex-mixed with 100 μl of 10% methanol water (1:9, v/v) and centrifuged at 12,000 rpm for 5 min, and then 80 μl of supernatant was transferred into a loading vial. The identification of different metabolites in our samples was conducted using Progenesis QITM (Waters, Milford, MA, United States), a software that enables the performance of peak alignment, peak selection, deconvolution, and identification of metabolites against HMDB library databases. Only samples with a mass accuracy within ±5 ppm and overall score ≥36 were regarded as highly confident and kept for subsequent analysis.

### Statistical and Metabolomic Analysis

Statistical analysis was performed using SPSS version 19 (IBM Inc., Armonk, NY, United States) and Prism version 8.0 (GraphPad Software Inc. San Diego, California, United States) for Windows. Student’s t test was applied to compare data with normal distribution among different data sets. The intergroup comparisons of non-normally distributed continuous variables were performed by means of the non-parametric Mann–Whitney *U* test to investigate the statistical characteristics. Median values and 95% confidence intervals (CIs) are described. For categorical variables, the χ^2^ test and Fisher’s exact test were used when appropriate. A *p* value <0.05 was regarded as statistically significant. The score plots of partial least squares–discriminant analysis (PLS-DA) with leave-one-out cross validation, quantitative enrichment analysis (QEA), receiver operating characteristic (ROC) curve analysis, univariate non-parametric Wilcoxon signed-rank test, and Spearman rank correlation analysis were performed using the online metabolomic analysis software MetaboAnalyst 5.0 (www.MetaboAnalyst.ca). Differential metabolites of each comparison were clustered according to their chemical structures in the heat map generated by the R package ggplot2. A network of discriminating metabolites and corresponding pathways was constructed based on the KEGG database by implementing Metscape (Gao et al., 2010), a plug-in installed in Cytoscape version 3.8.2.

## Results

### Participants Characteristics

#### Clinical Information About TTTS and FLC Surgery

Information related to TTTS and FLC, such as the gestational age, Quintero stage at diagnosis, surgery or delivery, and survival rates of FLC and EM groups, is presented in Supplementary Table S1. Notably, the gestational age (days) at diagnosis of TTTS in the EM group was much higher than that in the FLC-treated group (156.14 ± 24.24 vs 221.55 ± 23.65, *p* < 0.001), which may account for the larger proportion of FLC-treated cases diagnosed at earlier Quintero stages (7/14 at stage I in the FLC group vs 6/11 at stage III in the EM group). Within 2 days after the final diagnosis, FLC procedures were performed (at the 16th to 28th GWs) for the FLC group, according to published clinical guides (Middeldorp et al., 2007; Simpson and Simpson, 2013). For cases staged Quintero I in the FLC group, because of the short cervical length or severe maternal clinical manifestations, they also received FLC surgery, except for two cases in which the pregnancy was terminated by induced labor due to fetal cardiac anomalies, and the cases in the untreated group were delivered later than the 28th GW when diagnosed with TTTS; thus, it was not appropriate for them to undergo FLC procedures according to clinical guidelines, and they subsequently received cesarean section within 3 days after the TTTS diagnosis (Simpson and Simpson, 2013). There was no significant difference in the Quintero stage at diagnosis, gestational age at delivery, or survival rate in pregnancy between the FLC and Un groups. In addition, there was no significant difference in the amniotic deepest vertical pool in the recipients and donors of the FLC and Un groups at TTTS diagnosis (Supplementary Table S2) and in the larger and smaller co-twins between Con and PTB twin groups at the last ultrasound (Supplementary Table S3).

### Maternal Clinical Characteristics

The maternal demographic and clinical features of each subgroup and the significance of every comparison are listed in Supplementary Table S4. Maternal age, employment status, body mass index (BMI) before pregnancy or delivery, primigravida, smoking before or during pregnancy, conceptional mode, gestational age at delivery, delivery approaches, and neonatal sex were described and showed no significant difference in any of those comparisons (*p* > 0.05). The gestational age of the control group was markedly higher than that of any other group (*p* < 0.001), while there was no significant difference in any comparisons among the PTB, FLC, and Un groups.

### Neonatal Characteristics

Supplementary Table S5 presents the birth outcomes of each neonatal group in the present study. None of the comparisons showed a significant difference in placental weight or neonatal sex. Observably, the donor and recipient fetuses differed significantly in terms of birth weight, abdominal circumference, and amniotic fluid volume, while neither the body length nor head circumference significantly differed between the FLC and Un groups (C3 and C4). On the other hand, the inner group comparison of the Con and PTB groups did not demonstrate any significant differences in those variables (C1 and C2). The birth weight, body length, and head circumference of the Con group markedly outperformed those of the PTB, FLC, and Un groups (C5, C6, and C10). In addition, the abdominal circumference of the normal MCDA newborns was also higher than that of their FLC and Un counterparts (C5 and C6). Compared with the Con group, FLC (C7) and Un groups (C8) both showed a remarkably greater birth weight disparity and shorter body length and head circumference but presented no significant differences in parameters such as abdominal circumference, placenta weight, and newborn sex. Notably, the Un group had a significantly lower birth weight and greater discrepancy in amniotic fluid volume in C8, while the group of neonates who received FLC procedures did not differ from that in the PTB group in terms of these two variables, highlighting the greatly ameliorated birth outcomes of the FLC.

### PLS-DA Analysis of Placental Metabolomes Between Different Co-twin Groups in the Control, PTB, FLC, and Un Groups

The present study included 86 placental tissue samples collected from both co-twins in different groups, as shown in Figure 1. The placental metabolomic difference within co-twin pairs, in comparisons 1–4, was first investigated. As shown in Supplementary Figures S1A–D, when the larger/recipient co-twins and smaller/donor co-twins in the control, FLC, and Un groups were compared, the PLS-DA score plots indicated that the metabolomes in the placental tissue in those comparisons could not be clustered separately, which implies subtle metabolomic discrepancies within co-twin pairs between C1, C3, and C4.

**FIGURE 2:**
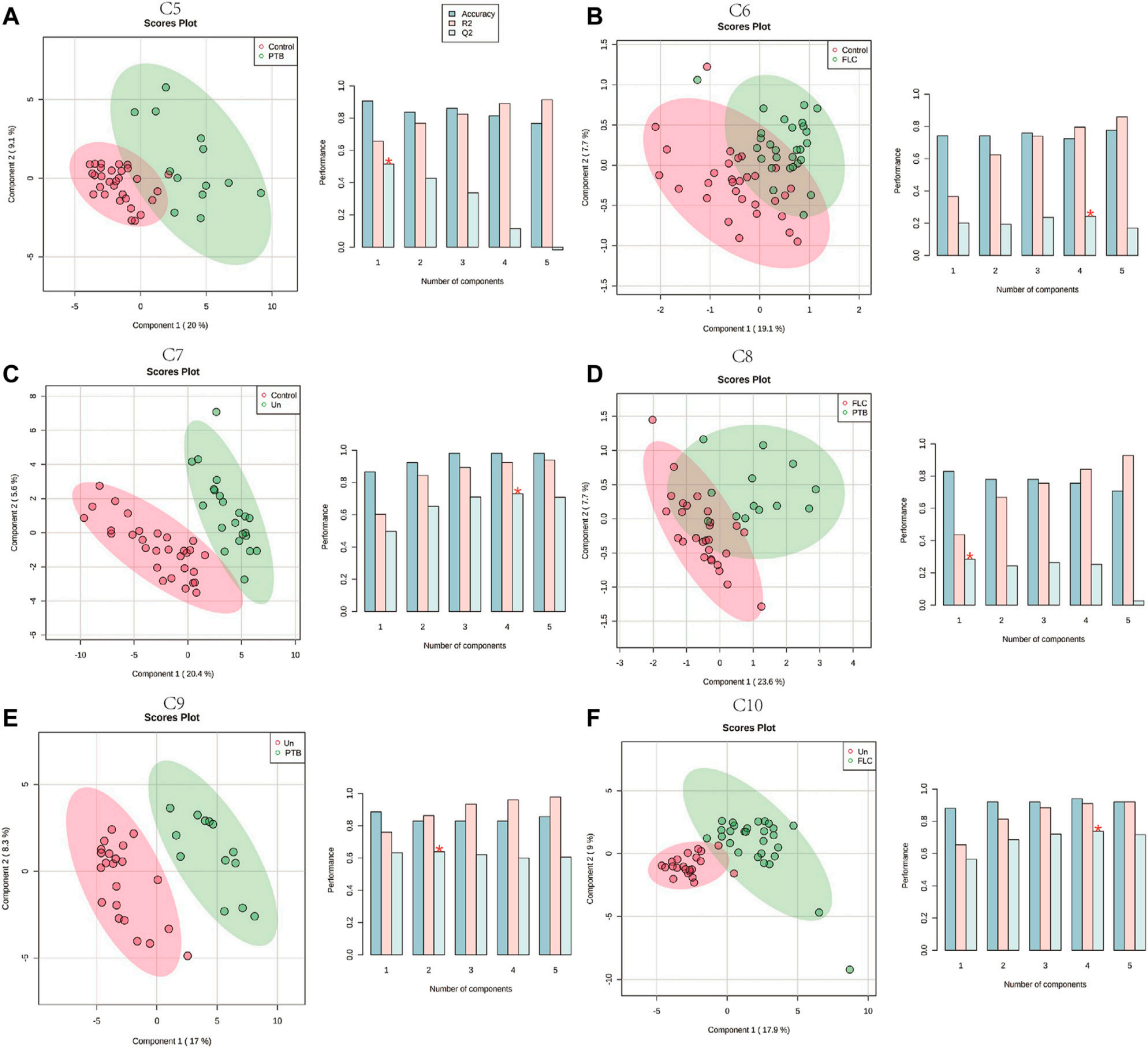
Partial least squares–discriminant analysis (PLS-DA) of the placental metabolome in comparisons 5–10. The left plots in **(A**–**F**) are the PLS-DA score plots of the corresponding comparisons. Histograms on the right display the evaluation of prediction model performance *via* leave-one-out cross validations (LOOCV), in which R2 represents the model’s explanatory ability of the data and Q2 reflects the predictive ability of the corresponding PLS-DA model.

Given that the placental metabolomes between different co-twins within the Con, PTB, FLC, and Un groups demonstrated little difference, we collectively grouped the larger/recipient or smaller/donor co-twins in each group and conducted intergroup comparisons (C5–C10). The PLS-DA score plots of C6 (Control vs FLC, Figure 2B) and C8 (PTB vs FLC, Figure 2D) were not distinctly separated, which implies that there were no significant metabolomic differences between those two comparisons. In other words, the FLC procedure might significantly mitigate the metabolomic disturbance of TTTS in the FLC group placenta, thus making it less different from the control and gestational age-matched PTB groups. However, when the placental metabolome of untreated TTTS pregnancies was compared to that of the control group (C7, Figure 2C), PTB group (C9, Figure 2E), and FLC-treated group (C10, Figure 2F), the PLS-DA displayed strikingly significant differences, suggesting that TTTS caused notable metabolomic changes in the placenta. Intriguingly, the score plot for C5, namely, the control group versus the PTB group, also showed an obvious separation, which is in accordance with the findings of previous studies that fetal and maternal metabolomes vary temporarily according to the gestational age (Ernst et al., 2020; Liang et al., 2020). Therefore, we focused mainly on gestational age-matched comparisons in the following analysis and compared the metabolomes of FLC donors, FLC recipients, Un donors, and Un recipients to the total number of PTB neonates (C11–C14, Supplementary Figures S1E–H). In addition, the comparison between the donors or recipients with or without the FLC procedure was carried out (C15 and C16, Supplementary Figures S1I,J). These six comparisons demonstrated clear separation and great performance.

### PLS-DA Analysis of Umbilical Cord Plasma Metabolomes Between Different Co-twin Groups in the Control, PTB, FLC, and Un Groups

**FIGURE 3:**
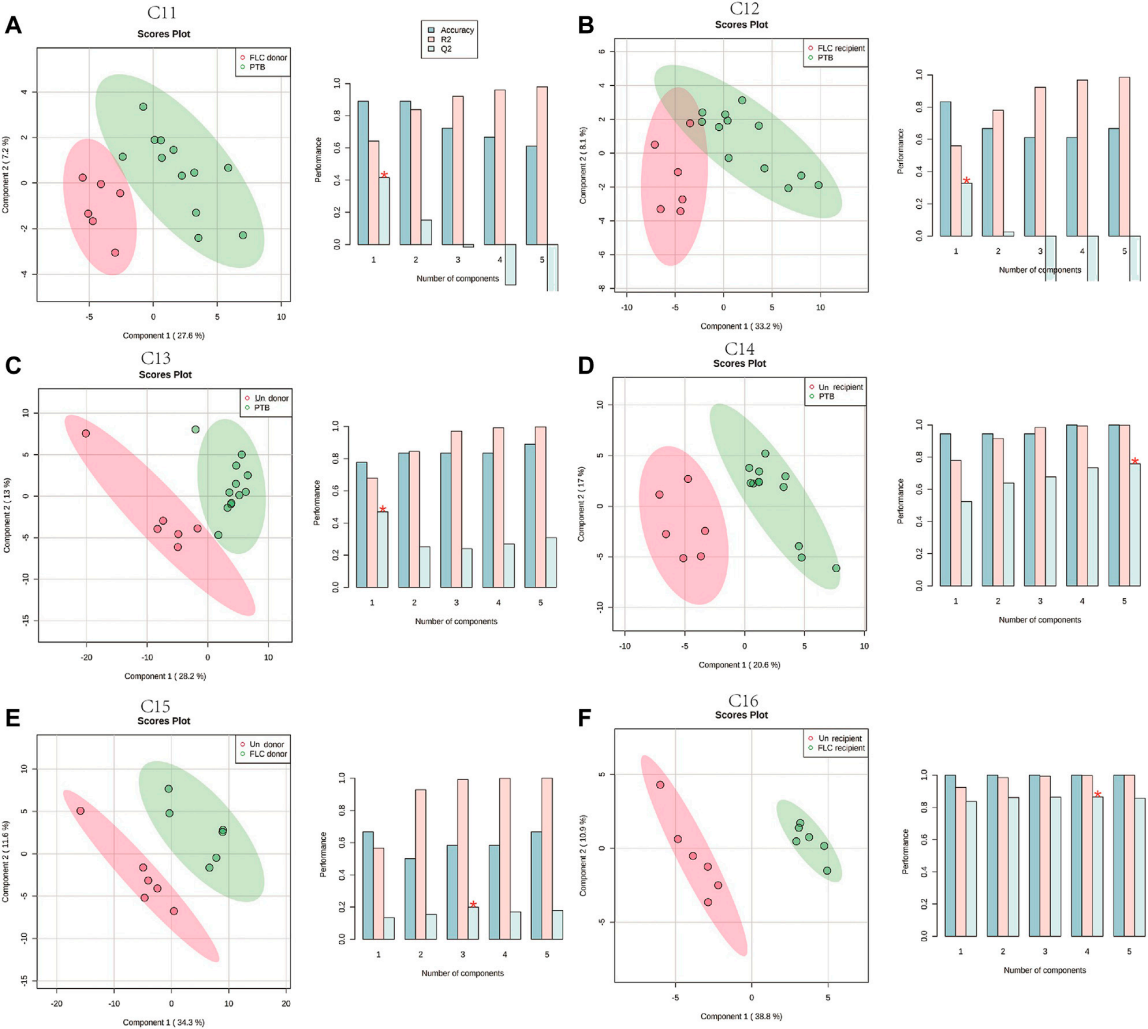
Partial least squares–discriminant analysis (PLS-DA) of the umbilical cord plasma metabolome in comparisons 11–16. The left plots in **(A**–**F)** are the PLS-DA score plots of corresponding comparisons. Histograms on the right display the evaluation of prediction model performance *via* leave-one-out cross validations (LOOCV), in which R2 represents the model’s explanatory ability of the data and Q2 reflects the predictive ability of the corresponding PLS-DA model.

In total, 48 cord plasma samples (six samples per subgroup) were detected using LC-MS in this study, and PLS-DA for the aforementioned 16 comparisons was subsequently performed. Unlike the placental metabolomes, the cord plasma samples showed a better capability for distinguishing the metabolomes of the different co-twin groups, as they clearly separated the metabolomes in the PLS-DA score plots of different co-twins in all the Con, PTB, FLC, and Un groups (Supplementary Figures S2A–D). When the major group comparisons were conducted, the cord plasma sample displayed a very similar result to the placental tissue. Specifically, the C5 (Con vs PTB, Supplementary Figure S2E), C7 (Con vs Un, Supplementary Figure S2G), C9 (Un vs PTB, Supplementary Figure S2I), and C10 (Un vs FLC, Supplementary Figure S2F) distinctly differentiated the “divisor” and “dividend” groups in the corresponding comparisons, and C6 (Con vs FLC, Supplementary Figure S2F) and C8 (FLC vs PTB, Supplementary Figure S2H) demonstrated a less clear separation. These findings indicated that 1) the gestational age is likely to also have a significant impact on the umbilical cord plasma metabolome (appearing in C5), 2) TTTS leads to some apparent metabolomic alteration in the cord plasma of the Un group (appearing in C7 and C9), and 3) FLC surgery greatly mitigates the metabolomic profile of cord plasma of the FLC group (appearing in C6, C8, and C10). To avoid the confounding effect of gestational age and to differentiate the metabolomic difference of the donors and recipients in the FLC and Un groups, we carried out C11–C16, which presented good separation (shown in Figure 3), indicating the existence of distinguishable metabolomic profiles between these co-twin groups.

### Pathway Analysis of Placental and Cord Plasma Metabolomes in the Comparisons of the Con, PTB, FLC, and Un Groups

In the PLS-DA, 55 discriminating metabolites were successfully matched with the corresponding compound superclass in the SMPDB (the Small Molecule Pathway Database, https://smpdb.ca/). There were 11 fatty acyls, 16 lipid- and lipid-like molecules, five organic acids, eight sterol lipids, and two sphingolipids found to be significantly different in C1–C10 in the placental metabolome (Supplementary Figure S3A). Regarding the umbilical cord plasma, 118 differential metabolites were matched with the corresponding superclass in the SMPDB. These discriminating metabolites in C1‐C16 in the umbilical cord plasma metabolome are 48 lipid and lipid-like molecules, 17 fatty acids, 15 sterol lipids, 5 prenol lipids, 4 organic acids, and 4 nucleic acids (Supplementary Figure 3B).

**FIGURE 4:**
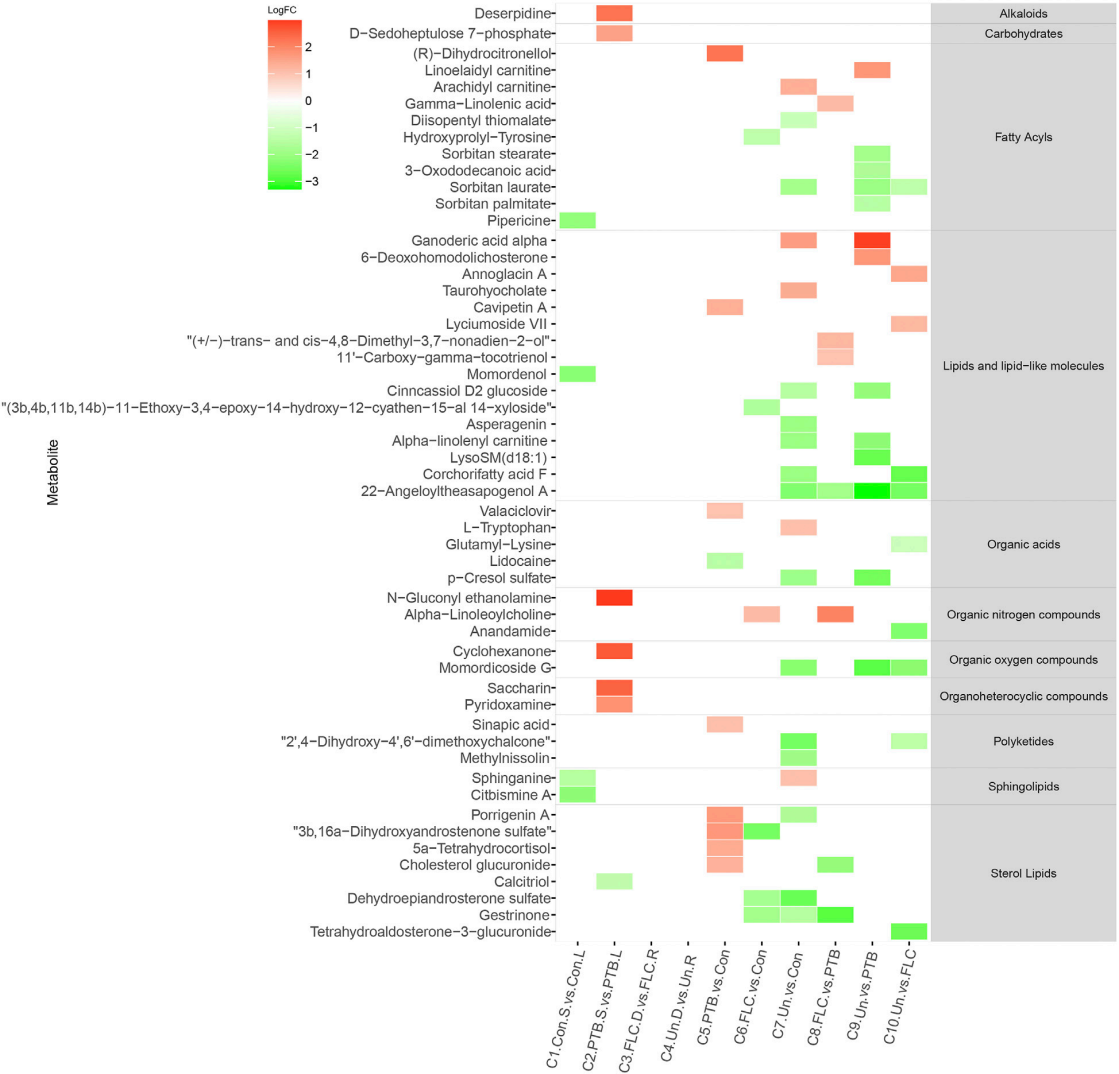
Heat map demonstrating the discriminating metabolites of corresponding comparisons and compound superclass in placental tissue metabolomes. The log_2_ scale matched with gradually changed colors shows the relative abundance of a given placental metabolite. Only differential metabolites (*p* value < 0.05, q value < 0.1, and FC > 2) that can be identified in the SMPDB and HMDB databases are displayed. Blocks with red or green represent higher or lower expression than the denominator group. Abbreviations: con, control; PTB, preterm birth; FLC, fetoscopic laser coagulation; Un, untreated; T, total; S, small; L, large; D, donor; R, recipient.

**FIGURE 5:**
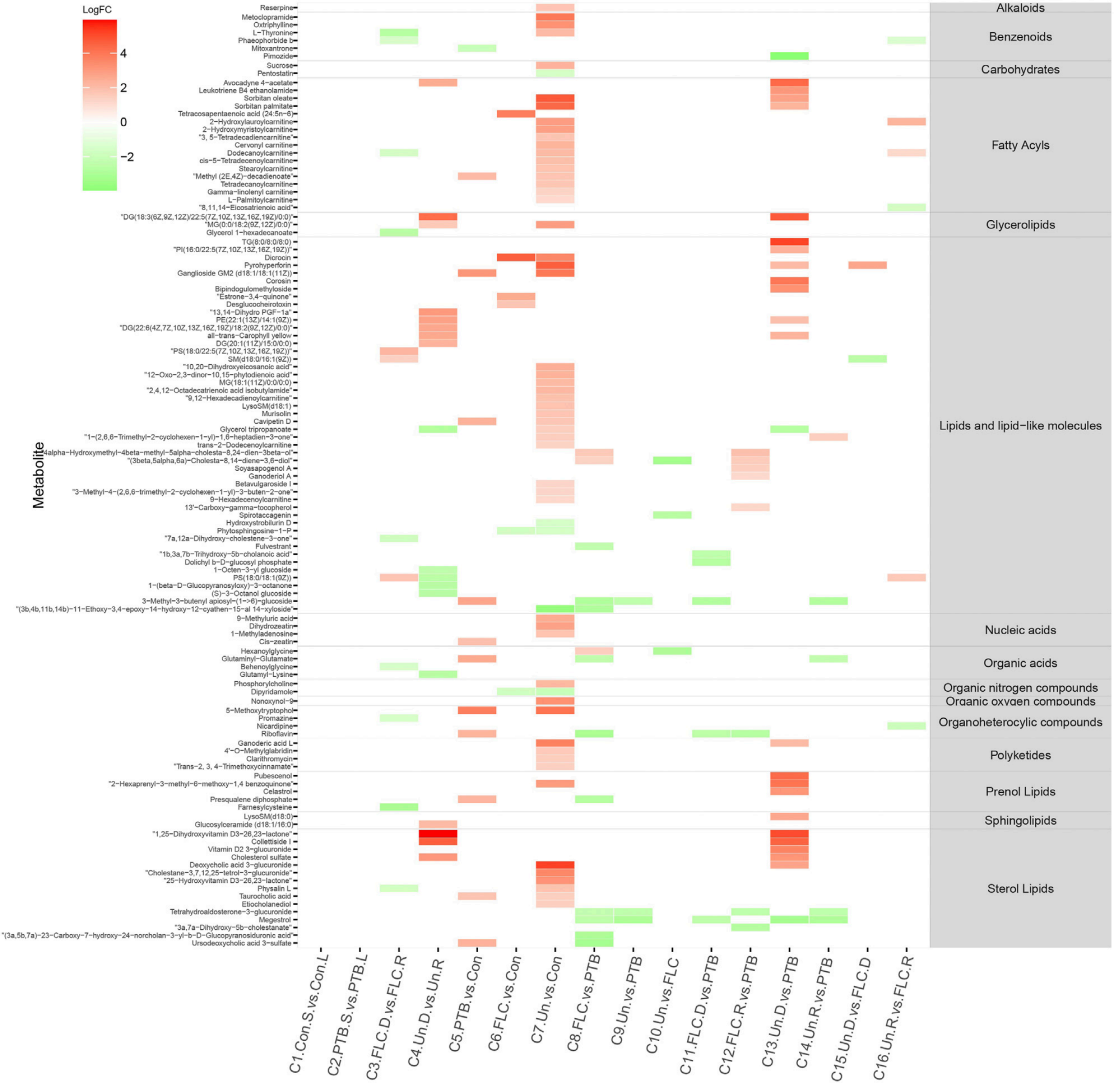
Heat map demonstrating the discriminating metabolites of corresponding comparisons and compound superclass in umbilical cord plasma metabolomes. The log_2_ scale matched with gradually changed color shows the relative abundance of a given placental metabolite. Only differential metabolites (*p* value < 0.05, q value < 0.1, and FC > 2) that can be identified in the SMPDB database are displayed. Blocks with red or green represent higher or lower expression than the denominator group. Abbreviations: con, control; PTB, preterm birth; FLC, fetoscopic laser coagulation; Un, untreated; T, total; S, small; L, large; D, donor; R, recipient.

We constructed a heat map of the significantly different metabolites (FC > 2, *p* value < 0.05, and FDR<0.1) in C1–C10 in the placental metabolomes and C1–C16 in the umbilical cord plasma metabolome according to their LogFC values. As shown in Figure 4, the donor and recipient co-twins had few differential placental metabolites in the FLC and Un groups (C3 and C4), whereas the major group comparisons depicted striking metabolomic discrepancies. Compared to the PTB group, the Un group showed decrease in four lipid- and lipid-like molecules, four fatty acyls, and one organic acid. Furthermore, compared to the FLC group, the Un group had lower levels of one organic acid, one sterol lipid, one fatty acyl, and two lipids and lipid-like molecules. On the other hand, as shown in Figure 5, the cord plasma revealed many discriminating metabolites in the intragroup comparisons of the FLC (C3) and EM groups (C4). Notably, the cord plasma of donors in the Un group displayed an increased enrichment in the levels of many sterol lipids, lipids and lipid-like molecules, and fatty acyls compared to the EM recipients and the PTB group, and this was not found in the FLC donors.

**FIGURE 6:**
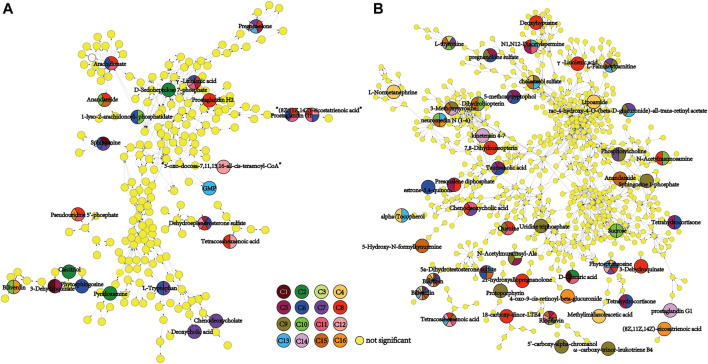
Metabolomic network of discriminating metabolic pathways in the 16 comparisons of placental **(A)** and umbilical cord plasma **(B)** metabolomes. The small yellow dots represent the non-significant metabolites in the pertinent pathways, and the colored dots represent the differential metabolites of the color-matched comparisons. Dots with multiple colors indicate that statistical significance was observed in all color-matched comparisons involved.

To depict the latent pertinent correlation between the differential metabolites in comparisons of the placental metabolome and umbilical cord plasma metabolome, metabolic networks were constructed (Figure 6). The levels of γ-linolenic acid, pregnenolone, dehydroepiandrosterone sulfate, and (8Z,11Z,14Z)-eicosatrienoic acid in the placental metabolome were significantly different among the multiple comparisons. Regarding the cord plasma metabolic network, the expression of phytosphingosine, tetracosahexaenoic acid, riboflavin, N1,N12-diacetylspermine, bilirubin, and biliverdin was found to be significantly different in many comparisons. Both networks shared similar annexes that center on sphingolipid, glycerophospholipid, linoleic acid, and steroid hormone metabolism.

**FIGURE 7:**
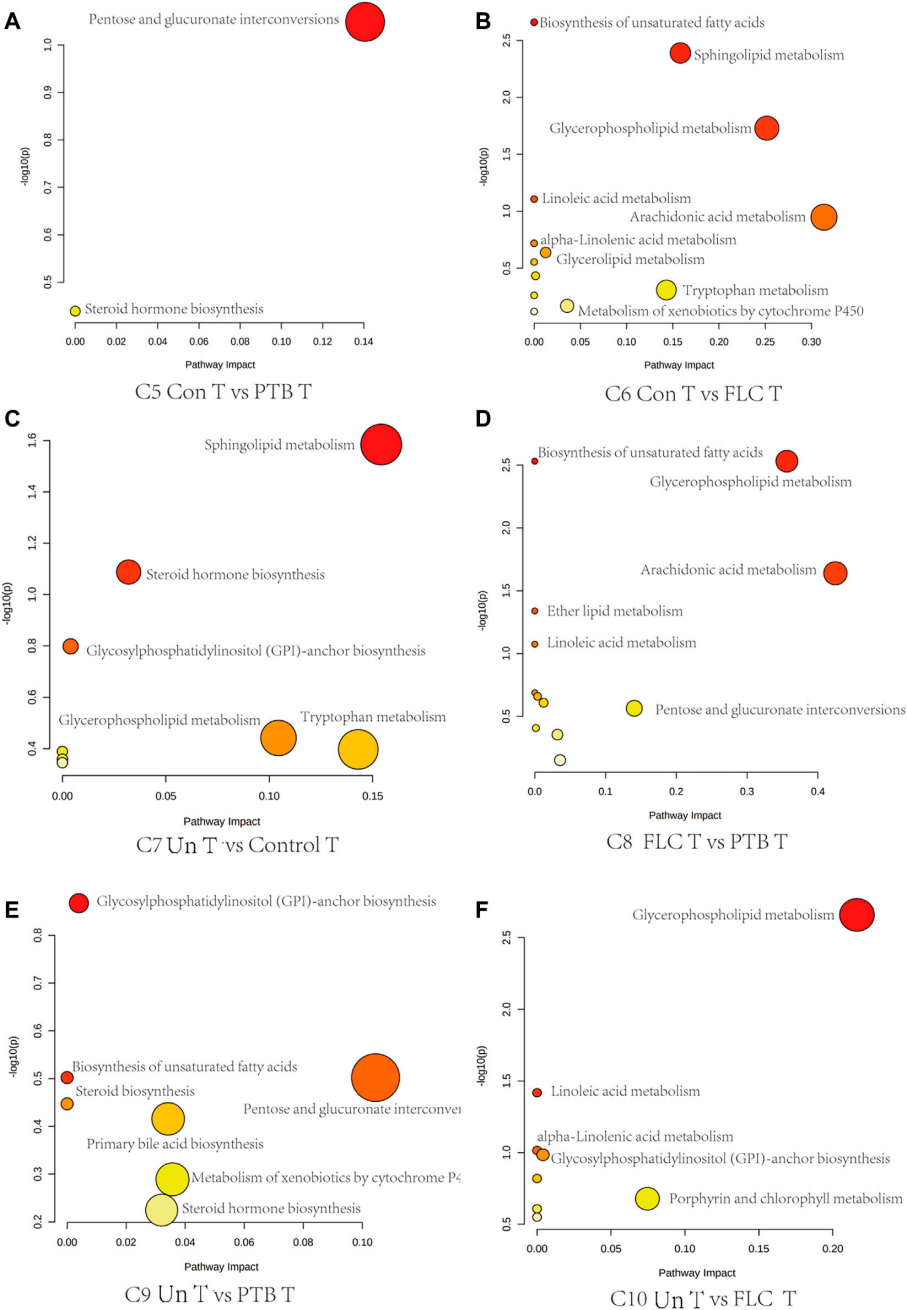
Bubble plots demonstrate the results of quantitative enrichment analysis of comparisons 5–10 in the placental metabolome. The significant metabolic pathways in corresponding comparisons are shown as bubbles in each plot. Bubbles with red and larger size indicate that they are more significant according to their p-values and pathway impact. Abbreviations: C, comparison; Con, control; PTB, preterm birth; FLC, fetoscopic laser coagulation; Un, untreated; T, total; S, small; L, large; D, donor; R, recipient.

**FIGURE 8:**
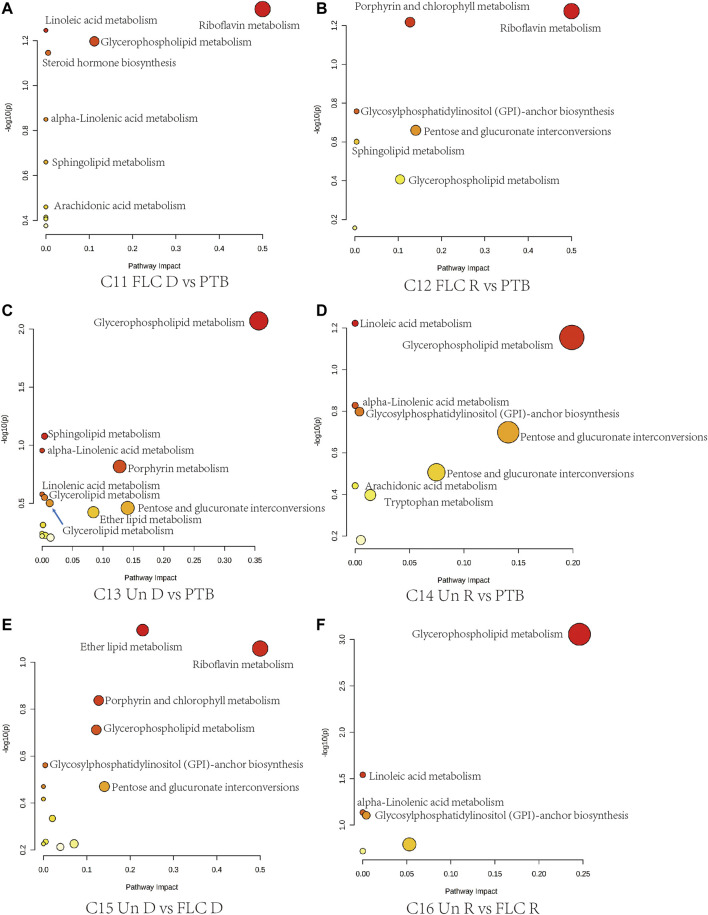
Bubble plots demonstrate the results of quantitative enrichment analysis of comparisons 11–16 in the umbilical cord plasma metabolome. The metabolic pathways with the most significance in corresponding comparisons are shown as bubbles in each plot. Bubbles with red and larger size indicate that they are more significant according to their p-values and pathway impact. Abbreviations: C, comparison; Con, control; PTB, preterm birth; FLC, fetoscopic laser coagulation; Un, untreated; T, total; S, small; L, large; D, donor; R, recipient.

Next, we conducted topological pathway analyses of each comparison of the placental and cord plasma metabolites. Based on the KEGG database, pertinent pathways of annotated discriminating metabolites (*p* value < 0.05, q value < 0.1, and FC > 2) in those comparisons are shown as bubbles in Figures 7, 8 and Supplementary Figure S4. In placental tissue, compared with the Con, PTB, and FLC groups, the Un group’s metabolome differed in terms of sphingolipid, glycerophospholipid, steroid, and unsaturated fatty acid metabolism (Figure 7), which is consistent with the results of the major group comparisons in umbilical cord plasma (Supplementary Figure S4). In our separate investigation of the discriminatory metabolic pathway in the umbilical plasma of donors and recipients in the Un and FLC groups compared to the PTB group, similar alterations in the metabolic pathways were observed; however, both the donors and recipients in the FLC group had altered riboflavin metabolism, which was also evident in the comparison of Un-D to FLC-D (Figure 8).

### Biomarker Analysis of Placental and Umbilical Cord Plasma Metabolites in Corresponding Comparisons

**FIGURE 9:**
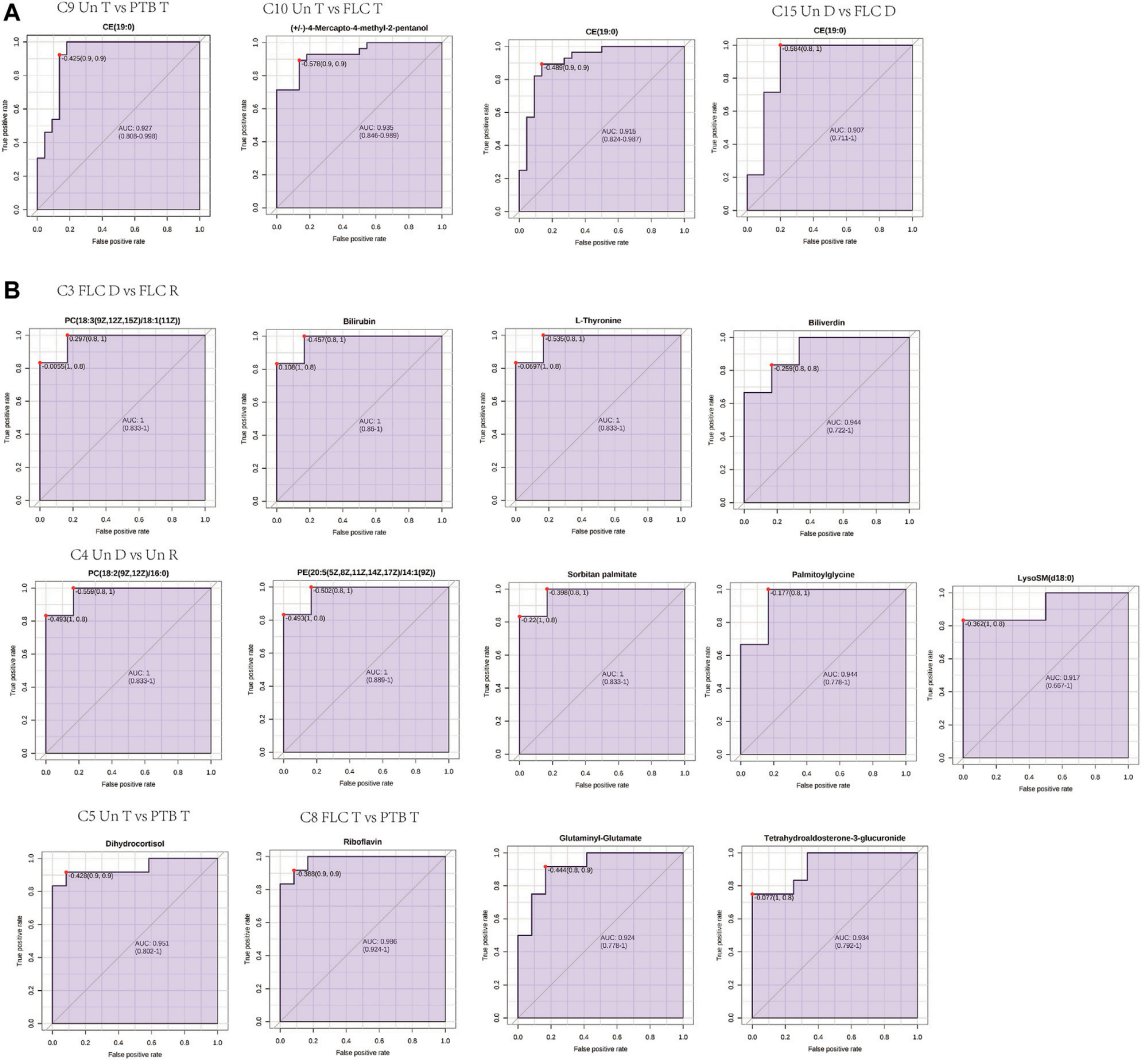
Receiver operating characteristic (ROC) curves of representative metabolites (AUC > 0.9) for corresponding comparisons in placental **(A)** and umbilical cord metabolomes **(B)**. Abbreviations: C, comparison; PTB, preterm birth; FLC, fetoscopic laser coagulation; Un, untreated; T, total; D, donor; R, recipient.

ROC curves for discriminating metabolites in each comparison of placental tissue and cord plasma were generated. Figure 9 displays representative images with an area under the curve (AUC) > 0.9. For placental comparisons, CE (19:0) was found to have good performance in C9, C10, and C15. Umbilical cord plasma metabolome comparisons had unveiled more potential biomarkers in more comparisons: C3-PC (18:3 (9Z,12Z,15Z) (16:0), bilirubin, l-thyronine, biliverdin; C4 PC (18:2 (9Z,12Z)/16:0), PE (20:5 (5Z,8Z,14Z,17Z (/14:1 (9Z)), sorbitan palmitate, palmitoyglycine, LysoSm (d18:0), C5 dihydrocortisol; C8 riboflavin, glutaminyl-glutamate, and tetrahydroaldosterone-3-glucuronide.

### Correlation Analysis of Discriminating Placental Metabolites to Neonatal Birth Weight and Ejection Fraction

We measured neonatal birth weight and LV-EF and correlated these parameters with placental differential metabolites in comparison 10 (Un vs FLC) *via* Spearman’s analysis. Metabolites with a p-value < 0.05 are shown in Table 1. (9Z,12Z,14E)-16-Hydroxy-9,12,14-octadecatrienoic acid was negatively correlated with birth weight, while SM(d18:1/18:1 (9Z)) was positively correlated with birth weight. Regarding the ejection fraction, six negatively correlated discriminating metabolites, namely, PE (20:2 (11Z,14Z)/15:0), 2-arachiodonylglycerol, PG (18:2 (9Z,12Z)/20:3 (8Z,11Z,14Z), CE (19:0), PE (15:0/14:0), and DG (15:0/0:0/14:1n5), were displayed.

**TABLE 1:** Spearman’s correlation analysis of placental differential metabolites with birth weight and cardiac function (ejection fraction).

Correlated parameters	Metabolite name	R	p-value
Birth weight	(9Z,12Z,14E)-16-Hydroxy-9,12,14-octadecatrienoic acid	−0.313	0.049
	SM(d18:1/18:1 (9Z))	0.338	0.014
Ejection fraction	PE (20:2 (11Z,14Z)/15:0)	−0.761	<0.001
	2-Arachidonyglycerol	−0.539	0.021
	PG (18:2 (9Z,12Z)/20:3 (8Z,11Z,14Z)	−0.528	0.024
	CE (19:0)	−0.640	0.004
	PE (15:0/14.0)	−0.582	0.011
	DG (15:0/0:0/14:1n5)	−0.545	0.019

## Discussion

TTTS is a highly fatal complication for both fetuses in MCDA pregnancy if left untreated. In recent decades, there have been various improvements in curative methods for TTTS, among which the FLC procedure is the most celebrated and effective one in mitigating birth outcomes. Although it is widely accepted that the pathological communication between different co-twins’ parts of the MCDA placenta *via* AVAs is the etiological basis for TTTS, the molecular mechanism that drives the sprouting and growth of AVAs remains ambiguous. Moreover, how FLC surgery improves the neonatal outcomes of newborns suffering from TTTS beyond the hemodynamic mechanism has rarely been reported.

By cross comparing the placental or umbilical cord plasma metabolomes of different co-twin groups, this research depicted a holistic metabolomic landscape of the placental tissue and umbilical cord plasma of TTTS pregnancies in the absence or presence of FLC procedures, revealing the different phenotypical metabolomic alterations in the donors and recipients of the Un group. We noticed that the FLC procedure greatly alleviated the metabolic disturbance for both co-twins and led to better neonatal outcomes, making their metabolomes less distinguishable from those of the control and PTB groups. FLC also mitigated birth weight discordance and improved the Apgar scores of FLC neonates. It was also demonstrated that intragroup comparisons of cord plasma metabolomes showed greater difference than the placental metabolomes, thus it can better differentiate the donors and recipients in the FLC and PTB groups. This finding, probably caused by the equalizing effect of vessel communication in the MCDA placenta, is consistent with our previous study on placental and cord plasma metabolomes of SIUGR (Wang et al., 2018).

The placental metabolomes also revealed that, compared with the control, PTB, and FLC groups, the untreated group had lower expression mainly of sterol lipids, fatty acyls, and lipids and lipid-like molecules, which are involved in sphingolipid metabolism, steroid hormone biosynthesis, glycerophospholipid metabolism, and biosynthesis of unsaturated fatty acids, among other metabolic processes. On the other hand, the cord plasma metabolomes indicated that the Un group also experienced perturbations in riboflavin metabolism and porphyrin metabolism compared to their counterparts in the FLC and PTB groups. Moreover, this study showed that among the placental metabolites, CE (19:0), a cholesteryl ester also known as cholesteryl nonadecanoic acid, has a strong diagnostic ability in distinguishing Un from PTB and FLC groups. In addition, glycerophospholipids such as PC (16:0/18:3 (9Z,12Z,15Z)), PC (18:2 (9Z,12Z)/16:0), PE (20:5 (5Z,8Z,14Z,17Z (/14:1 (9Z)), and sphingolipids such as LysoSm (d18:0) were all found to have high AUCs (>0.9) in discriminating donors and recipients in the Un or FLC group. By relating the differential metabolites with the birth weight and ejection fraction, we noticed that the cholesteryl ester CE (19:0) and glycerophospholipids including PE (15:0/14:0), PE (20:2 (11Z,14Z)/15:0), and PG (18:2 (9Z,12Z)/20:3 (8Z,11Z,14Z) were negatively correlated with ejection fraction and that SM (d18:1/18:1 (9Z)), a sphingolipid, was positively correlated with birth weight. Such a correlation between bound lipids and birth weight or neonatal echocardiographic measurements was also found in the cord plasma or amniotic fluid metabolome by many previous studies (Dunn et al., 2016; Hellmuth et al., 2017).

To date, evidence from previous studies that provides comprehensive insights into the metabolic aspects of TTTS has been exceedingly rare. Some studies have shed some light on certain types of metabolites or cytokines, such as iron, natriuretic peptides (Bajoria et al., 2002), circulating or placental angiogenic factors (Yinon et al., 2014; Chon et al., 2018), and amino acids (Bajoria et al., 2000). Until recently, by investigating the amniotic fluid metabolomes of the recipient co-twin of TTTS pregnancies that underwent FLC surgery, Dunn observed disrupted carbohydrate and fatty acid metabolism and found its correlation with fetal cardiovascular functions (Dunn et al., 2016). However, since the metabolites in the amniotic fluid could be secreted by the placenta or fetal organs and their secretion could be induced by trophoblast or vessel destruction in FLC surgery or amniodrainage, we still lack solid evidence to confirm the sources of these perturbed metabolites and their role in the pathogenesis of TTTS. Another newly published retrospective cohort study conducted by our team also reported disrupted amino acid and fatty acid metabolism in serum samples prospectively collected during the first gestational trimester of TTTS pregnancies (Yang et al., 2021).

However, there are no published articles on the holistic metabolomic profile of donor co-twins or recipient co-twins in TTTS-complicated umbilical cord plasma and placental tissue or the metabolic change brought by FLC surgery in those two types of samples. Kumazaki reported that the villi on the donor side of the TTTS-complicated monochorionic placenta have higher expression of pro-angiogenetic factors such as VEGF, Flt-1, and KDR (Kumazaki et al., 2002), implying that potential overactive angiogenesis may exist in the donor part of the TTTS placenta. Since the angiogenetic activity of endothelial cells mainly consumes energy substrates such as glucose, amino acids (AAs), and fatty acids (Bruning et al., 2018; Eelen et al., 2018), it would inevitably change the metabolomic profile of the TTTS placenta and even of the cord blood. For the fetus, the imbalanced circulation volume of both the donor and recipient co-twin, brought by the intertwining AVA, would also trigger the release or suppression of renin–angiotensin system (RAS) components and atrial natriuretic peptide (Mosquera et al., 2012), change the profile of fetal cardiac nutrients and oxygen supply for both co-twins (recipient heart receives too much blood, while donor gets deficient cardiac blood perfusion, nutrition, and oxygen supply), and cause cardiac anomalies in the recipients (hypervolemia leads to the hypertrophy and dilation of recipients’ heart) (Galea et al., 2005). Alternatively, in another hypothetical scenario, the metabolomic disparity could be the consequence of the TTTS or, more specifically, the AVAs, as data from both our group and others show that the FLC procedure brought significant changes to or improvements in the relative concentrations of hundreds of metabolites in the amniotic fluid, placental tissue or umbilical cord plasma (Dunn et al., 2016). Therefore, the metabolic clues in such tissues or fluids are of great significance for the elucidation of the etiological and pathophysiological machinery of TTTS.

In the present study, disrupted fatty acid metabolism was observed in untreated TTTS fetuses’ umbilical cord plasma and placental tissue, which is consistent with our previous finding in the later diagnosed TTTS maternal serum collected at early pregnancy (Yang et al., 2021). Compared with the FLC-treated group, the Un group had lower alpha-linolenic acid metabolism in placental metabolomes, and the recipients in the Un group also had less active biosynthesis of unsaturated fatty acids in the cord plasma metabolome. Even after the FLC procedures, the placental metabolomes of the FLC group still showed downregulated expression of arachidonic acid and linoleic acid metabolism compared to the PTB group. Many previous studies have reported the essential role of fatty acids in angiogenetic activities and the correlation of many adverse birth outcomes with disturbed fatty acid metabolism (Yang et al., 2018). Fatty acids can be transported and metabolized by endothelial cells (Eelen et al., 2018) and trophoblasts (Lewis et al., 2018), providing substrates and ATP for angiogenesis (Basak and Duttaroy, 2013) and facilitating the proliferation of human umbilical vein smooth muscle cells (Li et al., 2013) and tube formation by trophoblasts in early gestation (Johnsen et al., 2011). Moreover, essential fatty acids can also function as precursors for producing vasoactive substances for the fetus and influence fetal insulin sensitivity and neurodevelopment (Lewis et al., 2018), which may account for the pathogenesis of neural sequelae and endocrinal dysfunction in TTTS fetuses. Furthermore, in the setting of TTTS, the increased overload in recipients could lead to fetal cardiac prematurity (Pruetz et al., 2015), which may change the myocardium metabolic preference from carbohydrates to fatty acids (Lopaschuk et al., 1992), thus consuming more fatty acids and leading to phenotypic metabolomic differences.

Our findings provide novel insights into the pathophysiology of TTTS: metabolomic disturbance may play a role in the development of pathological AVA in the MCDA placenta, and the altered metabolic profile might be relevant to cardiac anomalies. These findings may not only present new clues for improving diagnostic methods of TTTS pregnancies but also promote refinement for current therapeutic approaches and even inspire novel pharmacological intervention for cases in which the besting timing of FLC surgery is missed. To investigate the correlation of differential metabolites and neurological or cardiac sequelae in neonates complicated by TTTS, further follow-up of the neonates in this study is needed. Moreover, although this research has pointed out that altered fatty acid, lipid, and lipid-like molecule metabolism is involved in the pathogenesis of TTTS, how these metabolic pathways impact the sprouting and growth of AVA and the hemodynamics of co-twins during pregnancy still lacks a clear explanation; thus, prospective animal experiments are needed to provide *in vivo* evidence.

## Conclusion

FLC surgery greatly mitigates the metabolomic disturbance brought by the TTTS in the placental tissue and cord plasma, which centers mainly on fatty acid and lipid-like molecule metabolism and thus makes the placental and cord plasma metabolomes of FLC-treated twins close to the control or PTB MCDA co-twins. Certain subsets of compounds, especially lipid derivatives, are helpful in differentiating co-twins with different hemodynamic statuses and are significantly correlated with birth weight or neonatal ejection fraction.

## Data Availability

The raw data supporting the conclusions of this article will be made available by the authors, without undue reservation.
