# Supplementary material for: Comprehensive Metabolomic Profiling of Cord Blood and Placental Tissue in Surviving Monochorionic Twins Complicated by Twin-Twin Transfusion Syndrome With or Without Fetoscopic Laser Coagulation Surgery: A Retrospective Cohort Study
Source: Front Bioeng Biotechnol. 2022 Apr 12;10:786755. doi: 10.3389/fbioe.2022.786755 (PMC9070302; doi:10.3389/fbioe.2022.786755)
Supplement: Supplementary file 1 [file DataSheet1.docx]

Supplementary Material

# Supplementary Tables

## Supplementary Table 1 TTTS and FLC related clinical characteristics of FLC treated or Untreated groups.

| Characteristics and pregnancy outcomes | FLC Treated group(n=14) | Untreated group(n=11) | P-Value |
| --- | --- | --- | --- |
| Gestational age at diagnosis of TTTS (days) ^a^ | 156.1±24.2 | 221.6±23.7 | ＜0.001 |
| Quintero Stage at diagnosis ^b^ |  | | 0.183 |
| I | 7/14 | 1/11 |  |
| II | 2/14 | 1/11 |  |
| III | 4/14 | 6/11 |  |
| IV | 1/14 | 2/11 |  |
| V | 0/14 | 1/11 |  |
| Gestational age when receiving FLC procedures (days) | 159.4±24.1 | -- | -- |
| Quintero Stage when receiving FLC |  |  | -- |
| I | 7/14 | -- | -- |
| II | 1/14 | -- | -- |
| III | 5/14 | -- | -- |
| IV | 1/14 | -- | -- |
| V | 0/14 | -- | -- |
| Quintero Stage at delivery |  |  | -- |
| I | -- | 1/11 | -- |
| II | -- | 1/11 | -- |
| III | -- | 6/11 | -- |
| IV | -- | 2/11 | -- |
| V | -- | 1/11 | -- |
| Gestational age at delivery(days) ^a^ | 237.9±16.6 | 224.8±23.9 | 0.131 |
| Survival at delivery ^b^ |  | | 0.420 |
| None fetus | 1/14 | 1/11 |  |
| Only 1 fetus | 0/14 | 2/11 |  |
| Both fetuses | 13/14 | 8/11 |  |

a Student’s T-test

b Fisher exact probability

## Supplementary Table 2 Amniotic deepest vertical pool (median (75-25^th^ centile)) of the recipients and donors in the FLC and Un group at the diagnosis of TTTS.

| Cotwin Group | FLC treated group  (n=14) | Untreated group  (n=11) | | P value |
| --- | --- | --- | --- | --- |
| Recipients | 117.0(140.0-91.0) mm | 157.0(163.0-144.0)mm | | 0.276^a^ |
| Donors | 8.5(16.0-2.0)mm | 7.0 (35.0-3.5)mm | 0.717^a^ | |

a Mann-Whitney U test

## Supplementary Table 3 Amniotic deepest vertical pool (median (75-25^th^ centile)) of larger and smaller cotwins in Con and PTB twins at last ultrasound monitoring.

| Cotwin Group | Control group(n=15) | Preterm birth group (n=7) | P value |
| --- | --- | --- | --- |
| Larger | 59.0(69.5-48.5)mm | 60.0(69.5-50.5)mm | 0.834^a^ |
| Smaller | 49.5(54.5-49.5)mm | 53.0 (56.5-49.0)mm | 0.367^a^ |
| Total | 54.0(60.0-45.5)mm | 54.0 (60.5-51.5)mm | 0.912^a^ |

a Mann-Whitney U test

| Characteristics and pregnancy outcomes | Control group  (n=15) | Preterm birth group  (n=7) | FLC Treated group  (n=13) | Untreated group  (n=8) | P-Value | | | | | |
| --- | --- | --- | --- | --- | --- | --- | --- | --- | --- | --- |
|  |  |  |  |  | C5 | C6 | C7 | C8 | C9 | C10 |
| Maternal age (years)^a^ | 27.9±4.5 | 28.3±4.2 | 27.2±4.6 | 26.6±3.5 | 0.687 | 0.482 | 0.623 | 0.120^a^ | 0.754^a^ | 0.863 |
| Gestational age(weeks) ^a^ | 37.3±0.5 | 34.3±1.6 | 33.9±2.3 | 33.4±2.1 | ＜0.001 | ＜0.001 | 0.744 | 0.400^b^ | 0.595^b^ | ＜0.001 |
| Education  (below high school) ^b^ | 2/15(86.7%) | 1/7(14.3%) | 1/13(7.7%) | 0/8（0%） | 1.000 | 0.526 | 1.000 | 0.467 | 1.000 | 000^c^ |
| Employed outside the home ^b^ |  | | | | 1.000 | 0.405 | 1.000 | 0.421^d^ | 1.000^c^ | 1.000^c^ |
| Yes | 13/15(86.7%) | 6/7(85.7%） | 12/1392.3%） | 8/8（100%） |  | | | | | |
| No | 2/15(13.3%) | 1/7（14.3%） | 1/13（7.7%） | 0/8（0%） |  |  |  |  |  |  |
| Pregestational Body mass index (kg/m^2^) ^a^ | 21.0±2.7 | 20.5±2.9 | 20.3±3.5 | 20.3±2.2 | 0.572 | 0.540 | 0.911 | 0.888^a^ | 0.990^a^ | 0.699 |
| Prenatal Body mass index (kg/m^2^) ^a^ | 27.6±2.4 | 26.6±1.9 | 26.7±3.5 | 27.9±2.1 | 0.426 | 0.782 | 0.964 | 0.246 | 0.389 | 0.355 |
| Primigravida ^b^ | 9/15(60.0%) | 5/7（71.4%） | 7/13（53.8%） | 4/8（50.0%） | 1.000 | 0.780 | 0.642 | 0.864^c^ | 1.000^c^ | 0.671 |
| Smoking before or during pregnancy ^b^ | 0/15(0%) | 0/7 (0%) | 0/13 (0%) | 0/8(0%) | 1.000^d^ | 1.000^d^ | 1.000^d^ | 1.000^d^ | 1.000^d^ | 1.000^d^ |
| Mode of conception ^b^ |  | | | | 1.000 | 1.000 | 0.589 | 0.421^d^ | 1.000^c^ | 0.545 |
| IVF-ET | 1/15(6.7%) | 1/7（14.3%） | 1/13（7.7%） | 0/8(0%) |  | | | | | |
| Natural conception | 14/15(93.3%) | 6/7（85.7%） | 12/13（92.3%） | 8/8(100%) |  |  |  |  |  |  |
| Delivery ^b^ |  | | | | 0.206 | 1.000 | 1.000 | 0.243^d^ | 0.505^c^ | 0.318 |
| Cesarean | 15（100%） | 6/7（85.7%） | 11/13(84.6%) | 8/8（100%） |  | | | | | |
| Vaginal | 0（0%） | 1/7（14.3%） | 2/13(15.4%) | 0/8（0%） |  |  |  |  |  |  |

**Supplementary Table 4 Maternal clinical characteristics.**

aStudent’s T-test

bMann-Whitney U test

cChi-square test.

dFisher’s exact test;

eIVF-ET, *In vitro* fertilization & embryo transfer.

*p < 0.001.

**Supplementary Table 5 Neonatal clinical characteristics.**

|  | Control group  (n=15) | | | Preterm birth group  (n=7) | | | FLC treated group  (n=13) | | | Untreated group  (n=8) | | | P-Value | | | | | | | | | |
| --- | --- | --- | --- | --- | --- | --- | --- | --- | --- | --- | --- | --- | --- | --- | --- | --- | --- | --- | --- | --- | --- | --- |
|  | L | S | T | L | S | T | R | D | T | R | D | T | C1 | C2 | C3 | C4 | C5 | C6 | C7 | C8 | C9 | C10 |
| Birthweight(g)^a^ | 2721±245.9 | 2586±221.4 | 2653±239.9 | 2079±455.5 | 1991±450.5 | 2035±437.6 | 1998±420.6 | 1571±493.1 | 1785±499.2 | 1816±334.2 | 1396±402.5 | 1606±418.1 | 0.1262 | 0.7252 | 0.0256 | 0.0395 | <0.0001 | <0.0001 | 0.1231 | 0.0105 | 0.2398 | <0.0001 |
| Birthweight discordance(g) ^a^ | 212.0±81.35 | | | 87.14±64.73 | | | 427.7±419.5 | | | 420.0±231.5 | | | -- | | | | 0.0614 | 0.0045 | 0.0493 | 0.0029 | 0.9628 | 0.0020 |
| Birthweight discordant ratio(%)^a^ | 7.748±3.058 | | | 4.226±3.130 | | | 20.93±19.12 | | | 23.72±14.44 | | | -- | | | | 0.0139 | 0.0004 | 0.0359 | 0.0040 | 0.7272 | 0.0213 |
| Body length(cm) ^a^ | 46.87  ±1.60 | 46.40  ±1.35 | 46.63±1.47 | 44.83±1.94 | 43.83±1.94 | 44.33±1.92 | 42.45±3.503 | 40.20±5.051 | 41.38±4.35 | 42.25±4.301 | 39.38±4.809 | 40.81±4.65 | 0.3952 | 0.3931 | 0.2456 | 0.2281 | <0.0001 | <0.0001 | 0.0341 | 0.0208 | 0.705 | 0.0002 |
| Head circumference(mm) ^a^ | 318.2±8.748 | 313.3±8.290 | 315.8±8.719 | 310.4±10.45 | 306.4±12.84 | 308.4±11.24 | 298.9±17.79 | 283.3±24.73 | 291.5±22.30 | 293.8±14.27 | 280.3±20.77 | 287.0±18.57 | 0.1538 | 0.6037 | 0.1108 | 0.1520 | <0.0001 | <0.0001 | 0.0320 | 0.0032 | 0.5205 | 0.0437 |
| Abdominal circumference(mm) ^a^ | 313.2±26.92 | 311.0±16.29 | 312.1±21.87 | 292.0±18.37 | 277.2±50.74 | 284.6±36.81 | 286.7±32.96 | 254.5±32.36 | 270.6±35.90 | 279.5±18.85 | 248.8±27.29 | 264.1±27.66 | 0.7944 | 0.5567 | 0.0312 | 0.0201 | <0.0001 | <0.0001 | 0.3180 | 0.1189 | 0.5513 | 0.0076 |
| Placenta weight(g) ^a^ | 661.4±95.66 | | | 738.0±133.10 | | | 678.5±91.46 | | | 635.0±55.55 | | | -- | | | | 0.6409 | 0.4851 | 0.2903 | 0.0745 | 0.2422 | 0.1822 |
| Amniotic fluid volume(ml) ^a^ | 580.0±227.4 | 493.3±171.0 | 536.7±202.5 | 480.0±83.67 | 360.0±230.2 | 420.0±175.1 | 525.5±96.06 | 409.1±122.1 | 467.3±122.6 | 2838±1632 | 193.8±221.1 | 1516±1769 | 0.2480 | 0.3052 | 0.0220 | 0.0005 | 0.1604 | 0.0042 | 0.3845 | 0.0085 | 0.0085 | 0.1120 |
| Neonatal sex^b^ |  | | | | | | | | | | | |  | | | | 0.295 | 0.364 | 1.000 | 1.000 | 1.000 | 0.747 |
| male | 16/30(53.3%) | | | 6/14(42.9%) | | | 10/26(38.5%) | | | 6/16(37.5%) | | |  | | | | | | | | | |
| female | 14/30(46.7%) | | | 8/14(57.1%) | | | 16/26(64.5%) | | | 10/16(61.9%) | | |  |  |  |  |  |  |  |  |  |  |
| Apgar Score |  | | | | | | | | | | | | | | | | | | | | | |
| at 1 min | 10 (9, 10) | 10 (9, 10) | 10 (9, 10) | 9 (8, 10) | 9 (8, 10) | 9 (8, 10) | 9 (8, 9) | 8 (4, 9) | 8 (6, 9) | 8 (6, 9) | 8 (4, 9) | 8 (5, 9) | 0.935 | 0.935 |  |  |  |  |  |  |  |  |
| at 5 min | 10 (10, 10) | 10 (10, 10) | 10 (10, 10) | 10 (10, 10) | 10 (9, 10) | 10 (9, 10) | 10 (9, 10) | 9(8, 10) | 10 (9, 10) | 9(8, 10) | 9(7, 10) | 9(8, 10) | 1.000 | 1.000 |  |  |  |  |  |  |  |  |
| at 10 min | 10 (10, 10) | 10 (10, 10) | 10 (10, 10) | 10 (10, 10) | 10 (10, 10) | 10 (10, 10) | 10 (9, 10) | 9(8, 10) | 10 (9, 10) | 9 (9, 10) | 9 (8, 10) | 9 (8, 10) | 1.000 | 1.000 |  |  |  |  |  |  |  |  |

aStudent’s T-test

bMann-Whitney U test

cChi-square test.

# Supplementary Figures
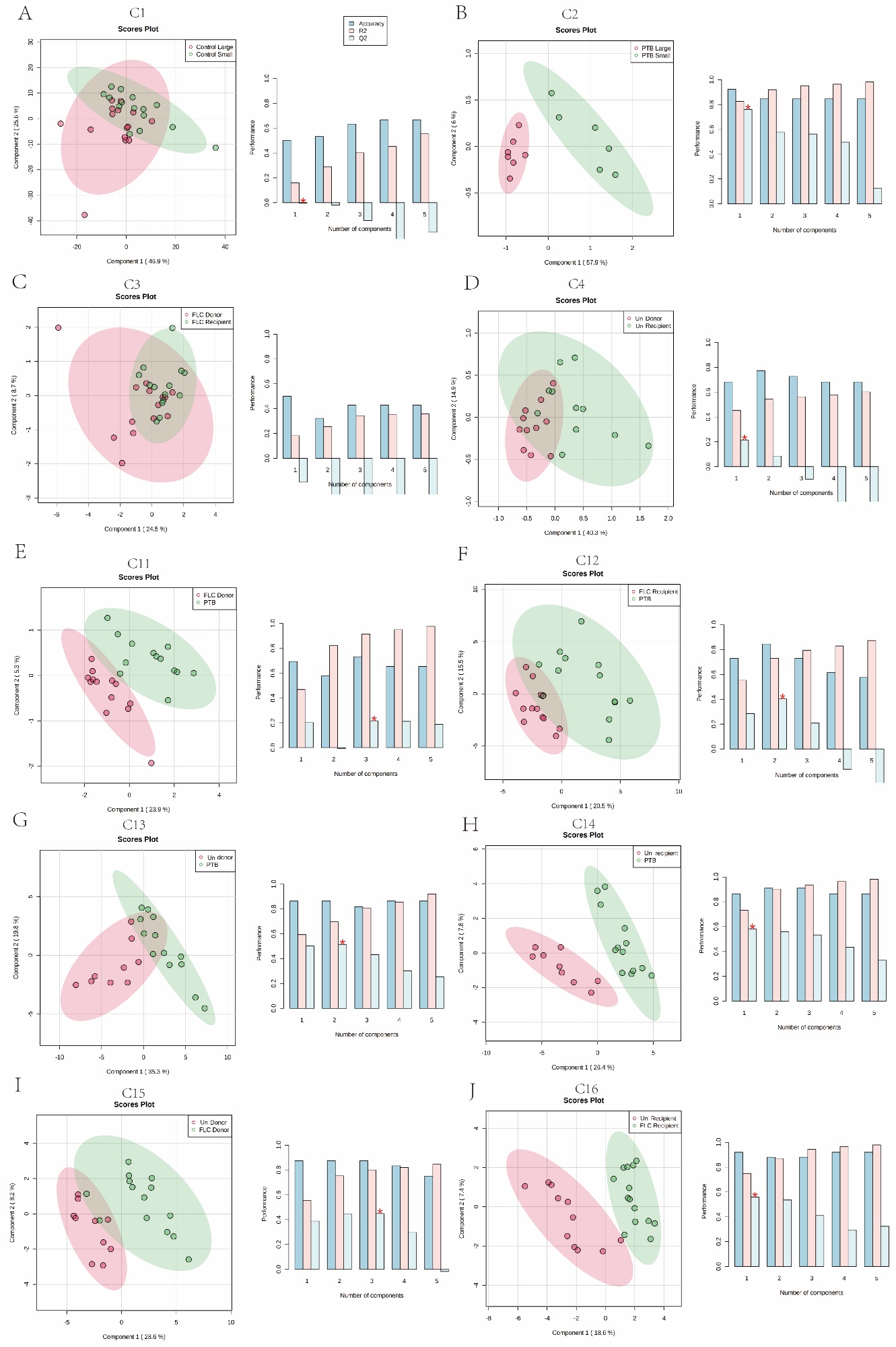


**Supplementary Figure 1. The partial least squares discriminant analysis (PLS-DA) of placental metabolome in comparison 1-4 and 11-16**

Left plots in **A-J** are the PLS-DA scores plots of corresponding comparisons. Histograms on the right display the evaluation of prediction model performance via leave-one-out cross validations (LOOCV) in which R2 represents the model’s explaining ability of data and Q2 reflexes the predictive ability of corresponding PLS-DA model.


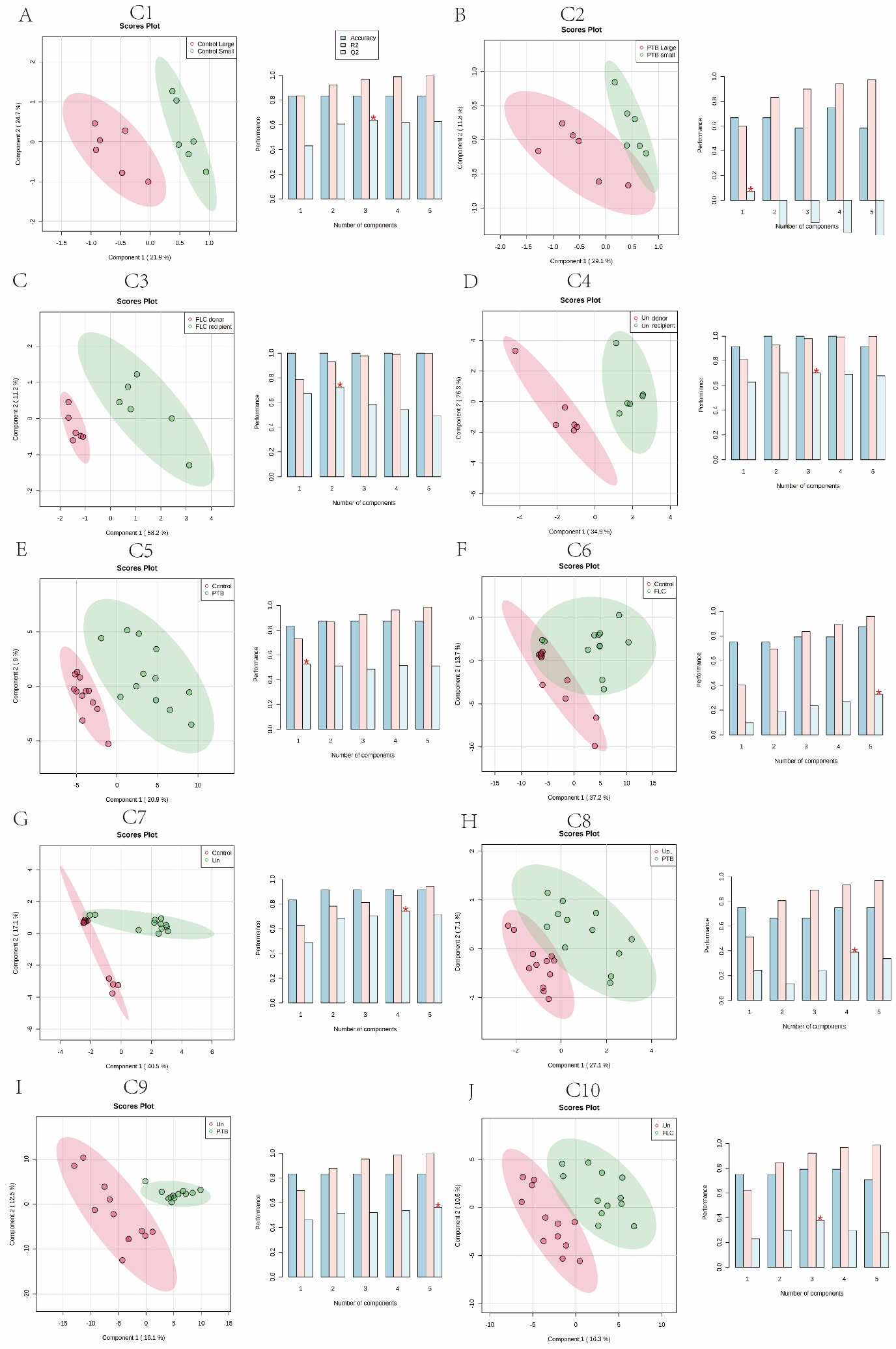


**Supplementary Figure 2. The partial least squares discriminant analysis (PLS-DA) of umbilical cord plasma metabolome in comparison 1 to 10**

Left plots in **A-J** are the PLS-DA scores plots of corresponding comparisons. Histograms on the right display the evaluation of prediction model performance via leave-one-out cross validations (LOOCV) in which R2 represents the model’s explaining ability of data and Q2 reflexes the predictive ability of corresponding PLS-DA model.

**
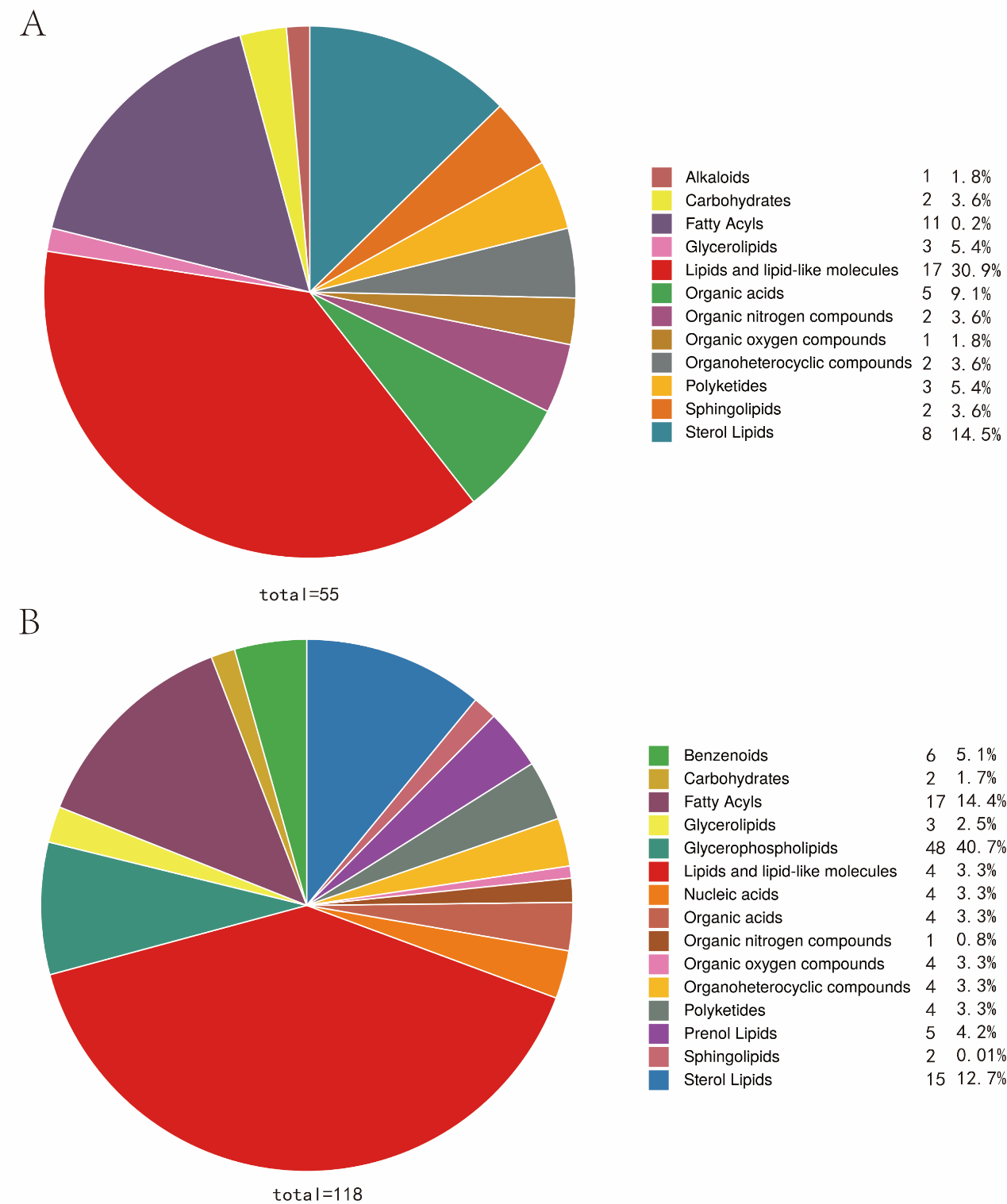
**

**Supplementary Figure 3. Pie chart demonstrates the ratio of different compound sets of discriminating metabolites in 16 comparisons of placental metabolomes (A) and umbilical cord plasma metabolome (B) according to the SPMDB database.**


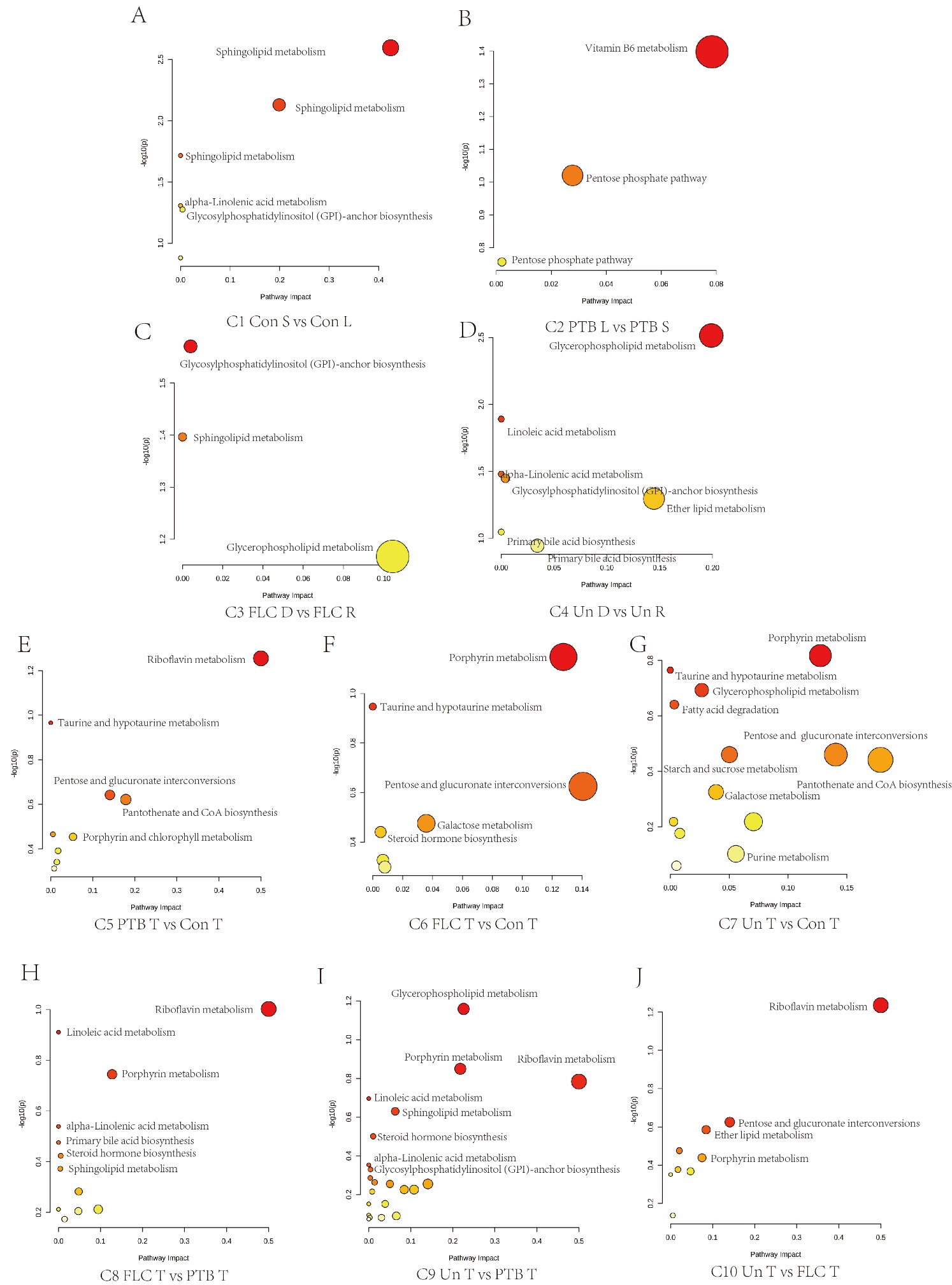


**Supplementary Figure 4. Bubble plots demonstrate the result of quantitative enrichment analysis of comparison 1 to 10 in umbilical cord plasma metabolome.** The metabolic pathways with most significance in corresponding comparisons are shown as bubbles in each plot. Bubbles with redder color and bigger size indicates they are more significant according to their p-value and pathway impact.
